# THC Reduces Ki67-Immunoreactive Cells Derived from Human Primary Glioblastoma in a GPR55-Dependent Manner

**DOI:** 10.3390/cancers13051064

**Published:** 2021-03-03

**Authors:** Marc Richard Kolbe, Tim Hohmann, Urszula Hohmann, Chalid Ghadban, Ken Mackie, Christin Zöller, Julian Prell, Jörg Illert, Christian Strauss, Faramarz Dehghani

**Affiliations:** 1 Department of Anatomy and Cell Biology, Medical Faculty of Martin-Luther University Halle-Wittenberg, Grosse Steinstrasse 52, 06108 Halle (Saale), Germany; marc.kolbe@medizin.uni-halle.de (M.R.K.); tim.hohmann@medizin.uni-halle.de (T.H.); urszula.hohmann@medizin.uni-halle.de (U.H.); chalid.ghadban@medizin.uni-halle.de (C.G.); 2 Department of Psychological & Brain Sciences, Indiana University, 1101E. 10th, Bloomington, IN 47405, USA; kmackie@indiana.edu; 3 Department of Neurosurgery, University Hospital Halle (Saale), Ernst-Grube-Str. 40, 06120 Halle (Saale), Germany; christin.zoeller@uk-halle.de (C.Z.); julian.prell@uk-halle.de (J.P.); joerg.illert@uk-halle.de (J.I.); christian.strauss@uk-halle.de (C.S.)

**Keywords:** brain tumor, glioblastoma, THC, CBD, cannabidiol, CB_1_, CB_2_, endocannabinoid system, GPR18, lysophosphatidylinositol, LPI

## Abstract

**Simple Summary:**

Glioblastoma (GBM) is the most frequent primary brain tumor entity with poor prognosis and resistance to current standard therapies. Cannabinoids, such as tetrahydrocannabinol (THC) and cannabidiol (CBD) are discussed as promising compounds for individualized treatment, as they exert anti-tumor effects by binding to cannabinoid-specific receptors. However, their pharmacology is highly diverse and complex. The present study was designed to verify (1) whether cannabinoids show even any effect in GBM cells derived from primary human tumor samples and (2) to identify the receptor responsible for those effects. Our findings revealed that THC reduces the number of Ki67 immunoreactive nuclei, a cell cycle marker through the orphan cannabinoid receptor GPR55. The data suggest a therapeutic potential of cannabinoids in those GBM with functional and responsive GPR55.

**Abstract:**

Glioblastoma (GBM) is the most frequent malignant tumor of the central nervous system in humans with a median survival time of less than 15 months. ∆^9^-Tetrahydrocannabinol (THC) and cannabidiol (CBD) are the best-characterized components of *Cannabis sativa* plants with modulating effects on cannabinoid receptors 1 and 2 (CB_1_ and CB_2_) and on orphan receptors such as GPR18 or GPR55. Previous studies have demonstrated anti-tumorigenic effects of THC and CBD in several tumor entities including GBM, mostly mediated via CB_1_ or CB_2_. In this study, we investigated the non-CB_1_/CB_2_ effects of THC on the cell cycle of GBM cells isolated from human tumor samples. Cell cycle entry was measured after 24 h upon exposure by immunocytochemical analysis of Ki67 as proliferation marker. The Ki67-reducing effect of THC was abolished in the presence of CBD, whereas CBD alone did not cause any changes. To identify the responsible receptor for THC effects, we first characterized the cells regarding their expression of different cannabinoid receptors: CB_1_, CB_2_, GPR18, and GPR55. Secondly, the receptors were pharmacologically blocked by application of their selective antagonists AM281, AM630, O-1918, and CID16020046 (CID), respectively. All examined cells expressed the receptors, but only in presence of the GPR55 antagonist CID was the THC effect diminished. Stimulation with the GPR55 agonist lysophosphatidylinositol (LPI) revealed similar effects as obtained for THC. The LPI effects were also inhibited by CBD and CID, confirming a participation of GPR55 and suggesting its involvement in modifying the cell cycle of patient-derived GBM cells.

## 1. Introduction

Glioblastoma (GBM) is the most devastating form of primary brain tumors in adults, with an extremely poor prognosis [1]. The poor success of the current standard modalities consisting of neurosurgical resection followed by an adjuvant radio- and chemotherapy is attributed to the immense radio- and chemoresistance and the remarkable heterogeneity between and within individual tumors [2]. This heterogeneity is reflected by different cells-of-origin that exhibit distinct phenotypic and molecular characteristics and different behavior in terms of therapy sensitivity, tumorigenicity, proliferation, motility, invasiveness, genetics, and epigenetics [2,3]. Accordingly, due to the presence of such a heterogeneous GBM cell population, new and individualized therapeutic strategies are urgently needed.

In recent years, the human endocannabinoid system (ECS) has been identified as a promising and novel therapeutic target for treatment of various tumor forms. The ECS consists of a series of natural ligands, the associated enzyme machinery (for biosynthesis, degradation, and transport), and specific cannabinoid receptors [4]. Thus far, two cannabinoid-specific receptors have been identified: the CB_1_ and CB_2_ receptors [5,6]. Intriguingly, these receptors have been described in human tumors from lung, breast, prostate, skin, pancreas, lymphoma, and brain [7,8,9,10,11]. By activating CB_1_ or CB_2_, different phyto- and synthetic cannabinoids were previously shown to exert anti-tumoral effects, leading to apoptosis or preventing proliferation, invasion, and angiogenesis [9,11,12,13,14,15,16].

In particular, ∆^9^-tetrahydrocannabinol (THC) and cannabidiol (CBD), the main ingredients of *Cannabis sativa* plants, caused a significant decrease in cell viability and proliferation in a variety of GBM cell lines, including SF126, U251, U87, C6, and also primary GBM cells by cell cycle arrest or apoptosis [12,13,14]. Growth inhibition of U87-MG cell-based tumor xenografts has been reported after THC and CBD exposure by autophagy-mediated apoptosis [14]. Furthermore, CBD has been shown to inhibit the invasion of U87-MG and T98G cells [13]. Remarkably, CBD potentiates the anti-tumor effects of THC apparently via different receptors and signaling pathways [12,14]. It was demonstrated that THC effects were often triggered by CB_1_ and CB_2_ receptor activation, but the receptor engaged by CBD was mostly not clarified [14]. Indeed, THC shows a high agonistic activity on CB_1_ and CB_2_, while CBD binds to both receptors with a very low binding affinity [17,18]. Additionally, THC and CBD also modulate certain orphan receptors, such as GPR55 and GPR18, ion channels, transient receptor potential (TRP) channels in particular, or peroxisome proliferator-activated receptors (PPAR) such as PPARγ [19,20,21].

Among these receptors, GPR55 should be highlighted as a putative “third” cannabinoid receptor, as it might account for some previously reported non-CB_1_/CB_2_ effects of THC and CBD [10,14]. Most evidence has consistently shown that GPR55 is activated by the endogenous phospholipid L-α-lysophosphatidylinositol (LPI) [22], and a crucial role of LPI in tumor progression was postulated. Thus, GPR55 was detectable in a number of tumor entities including melanoma, colon, breast, cholangiocarcinoma, prostate cancer, and GBM [23,24,25,26]. Since GPR55 promotes proliferation, migration, invasion, and metastasis of different tumor cells, its expression has been correlated with tumor aggressiveness; higher histological grades; and, in the case of gliomas, lower survival rates [23]. Despite the fact that GPR55 is phylogenetically distinct from CB_1_ and CB_2_ and displays a low sequence identity with both (13% and 14%, respectively) [27], several groups proposed an engagement of GPR55 by several cannabinoids including THC and CBD [19,26]. However, the heterogeneous interplay of THC and CBD with multiple targets contributes to a much more complex picture of their pharmacology and gives rise to the need for a detailed and individual characterization of target receptors involved in cannabinoid-mediated anti-tumor effects.

The aim of this study was to examine the receptor behind the impact of THC and CBD on the cell cycle by Ki67-staining of patient-derived tumor cells of human GBMs. These cells were first characterized regarding their expression pattern of different receptors that are potential targets of both cannabinoids. Afterwards, pharmacological analyses with different receptor-specific agonists and antagonists were performed to illustrate the receptor behind the reduction of Ki67-immunoreactive cells after THC exposure. The obtained results suggest that the effects of THC are related to an activation of GPR55.

## 2. Results

### 2.1. GBM Cells Differed in Their Morphology and Showed High Proliferation Capacity

After isolation of the patient-derived GBM cells, their morphology and proliferation capacity were assessed.

**Figure 1:**
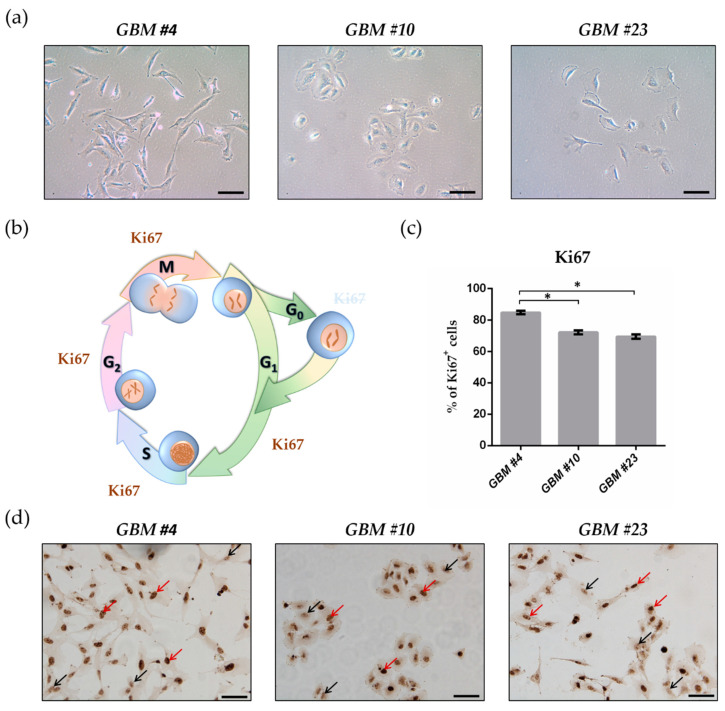
Morphological features and Ki67 immunoreaction of patient-derived glioblastoma (GBM) cells. (**a**) Representative light microscope images of the cell morphology of *GBM #4*, *GBM #10*, and *GBM #23*. Scale bar = 50 µm. (**b**) Schematic overview showing the presence of Ki67 during the cell cycle. Ki67 is located in the nucleus and is detectable during the whole cell cycle (G_1_-, S-, G_2_-, and M-phase), except for the G_0_-phase. (**c**) Graph indicates the percentage of GBM cells showing Ki67 positivity (Ki67^+^). Patient-derived GBM cells exhibited different and high numbers of Ki67-labbelled cells. Their percentage was as follows: 84.73% ± 1.11% (*GBM #4*, *n* = 34), 72.12% ± 1.18% (*GBM #10*, *n* = 32), and 69.42% ± 1.44% (*GBM #23*, *n* = 22). Data are means ± SEM of all untreated control groups of this study. Significance was chosen for *p* < 0.05. The asterisk denotes significant results regarding the respective measurement indicated with the bar. (**d**) Representative images of Ki67-stained *GBM #4*, *GBM #10*, and *GBM #23*. Brown staining represents Ki67^+^ cells (red arrows) and blue staining Ki67^-^ cells (black arrows). Nuclei were counterstained using hematoxylin. The number of values is represented by *n*. Scale bar = 75 µm.

All isolated tumor cells grew adherently as monolayer. Under the light microscope, a morphological heterogeneity between the tumor cells of the three individual tumors was observed (Figure 1a). In particular, *GBM #4* exhibited an elongated mesenchymal-like phenotype, whereas *GBM #10* had a round or elliptical epithelial-like morphology (Figure 1a). *GBM #23* was variable from rather round to elongated shaped (Figure 1a).

In general, the examined cells showed high but different numbers of Ki67-immunoreactive fractions. *GBM #4* displayed the highest fraction of about 85%, whereas in *GBM #10* and *GBM #23*, Ki67 was expressed by approximately 72% and 69% of the cells, respectively (Figure 1c,d).

Further molecular characteristics (MGMT promotor status, IDH1 mutation status, and expression of stem cell marker) were determined (Table S1).

### 2.2. THC Decreased Ki67^+^ Cells and Was Blocked by CBD

**Figure 2:**
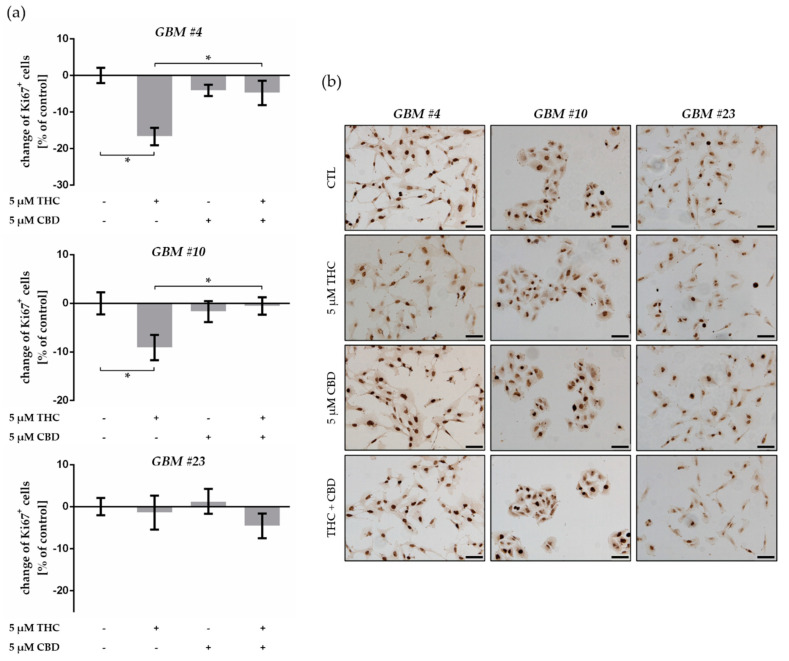
Impact of ∆^9^-tetrahydrocannabinol (THC) and cannabidiol (CBD) on proliferation capacity of human patient-derived GBM cells. (**a**) Effects of THC and CBD on the number of Ki67-labelled GBM cells. In comparison to control group, application of THC (5 µM) for 24 h produced a significant reduced number of Ki67^+^ cells in *GBM #4* and *GBM #10,* but not in *GBM #23*. Alterations after treatment with CBD (5 µM) were not observed, but in combination with THC it reversed the effects of THC in *GBM #4* and *GBM #10*. Data are means ± SEM of *N* = 3 independent experiments performed in duplicate. Significance was chosen for *p* < 0.05. The asterisk denotes significant results regarding the respective measurement indicated with the bar. (**b**) Representative images of Ki67-immunoreactive GBM cells (Ki67^+^ in brown and Ki67^-^ in blue) untreated (CTL) or treated with THC (5 µM), CBD (5 µM), and an equimolar combination of THC and CBD after 24 h. Nuclei were counterstained using hematoxylin. Scale bar = 75 µm.

The effects of cannabinoids on the number of patient-derived GBM cells entering the cell cycle were measured by Ki67 after treatment with THC and CBD. We found a significant decrease in the number of Ki67-immunoreactive nuclei after application of 5 µM THC after 24 h in *GBM #4* and *GBM #10*, but not in *GBM #23* (Figure 2, Table S2). In addition, stimulation with 5 µM CBD did not cause any changes in all examined GBM cells (Figure 2, Table S2). After simultaneous application of THC and CBD, CBD reversed the effects of THC in both *GBM #4* and *GBM #10* (Figure 2, Table S2).

### 2.3. GBM Cells Expressed Classical and Orphan Cannabinoid Receptors

**Figure 3:**
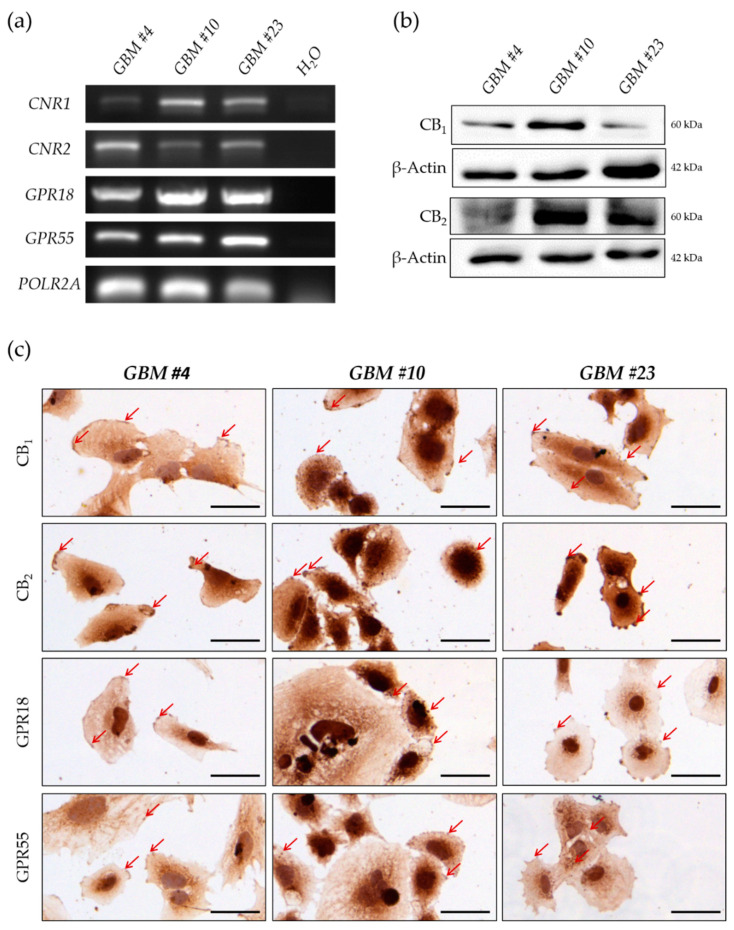
Characterization of human GBM cells regarding their expression of classical and orphan cannabinoid receptors. (**a**) mRNA for human CB_1_ (*CNR1*), CB_2_ (*CNR2*), GPR18 (*GPR18*), and GPR55 (*GPR55*) receptors were present in all GBM cells. RNA polymerase II subunit A (*POLR2A*) was used as a housekeeping gene. (**b**) Western blot analysis revealed the expression of all receptors on protein level. β-Actin was used as loading control. (**c**) Immunochemical staining showing the presence of CB_1_, CB_2_, GPR18, and GPR55 on the cell surface of *GBM #4*, *GBM #10*, and *GBM #23* (red arrows). Nuclei were counterstained using hematoxylin. Scale bar = 50 µm.

To identify the involved receptor reducing the number of Ki67^+^ cells after THC exposure, we examined the expression of different classical (CB_1_ and CB_2_) and orphan cannabinoid receptors (GPR18 and GPR55). PCR analysis revealed the expression of all receptors in every patient-derived cell type (Figure 3a and Figure S1a). Consistent with these data, CB_1_, CB_2_, GPR18, and GPR55 were detectable at the protein level in *GBM #4*, *GBM #10*, and *GBM #23*, as found in Western blot analyses or immunochemical staining (Figure 3b,c and Figure S1b). Subcellularly, all examined receptors were localized on the cell membrane, as reflected by the strong brown staining of the cell membrane with a partly punctuated staining pattern and markedly stained cell protrusions (Figure 3c). Notably, CB_2_ receptor showed a more intense immunostaining in *GBM #23* when compared either to *GBM #4* and *GBM #10* or to the immunostaining of the other receptors in the same cells (Figure 3c).

### 2.4. THC-Dependent Reduction of Ki67^+^ Cells Was Not Mediated by CB_1_, CB_2_, and GPR18

**Figure 4:**
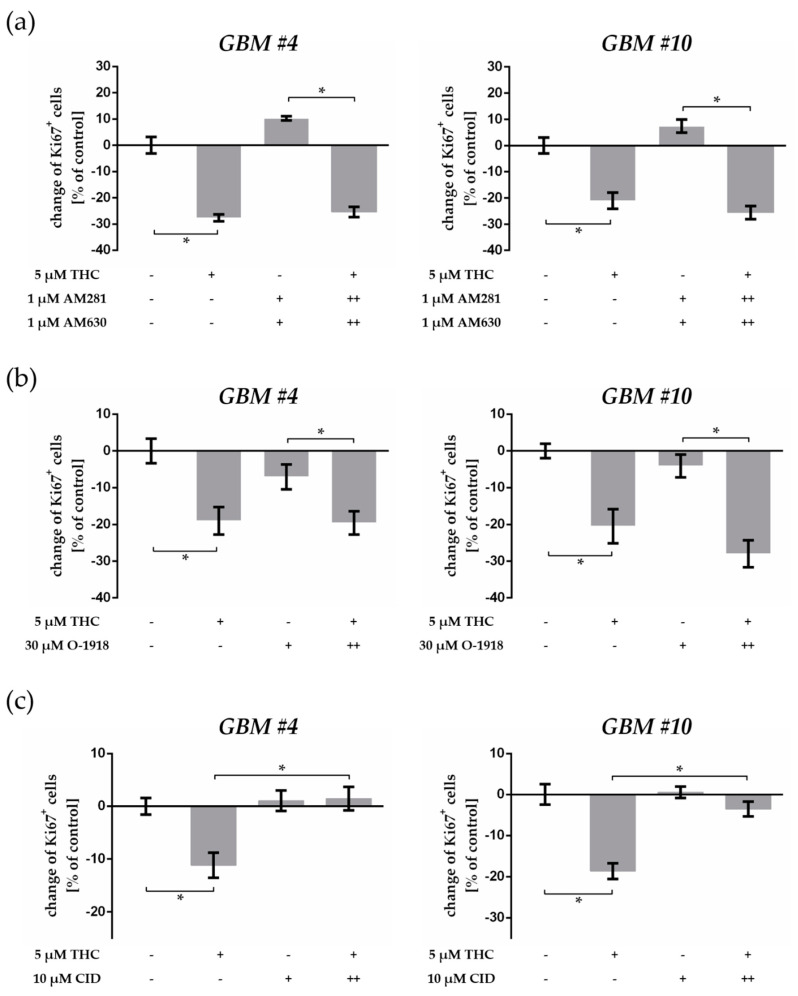
Impact of receptor-specific antagonists on THC-triggered effects. (**a**) Impact of CB_1_- and CB_2_-antagonists AM281 and AM630 on THC effects. AM281 (1 µM) and AM630 (1 µM) combined with THC (5 µM) had no effects on the decreased number of Ki67^+^ cells by THC. Co-stimulation with AM281 and AM630 alone did not influence the percentage of Ki67^+^ cells in G*BM #4* and *GBM #10* after 24 h compared to the control group. (**b**) Impact of GPR18 antagonist O-1918 on THC effects. After inhibition of GPR18 by O-1918 (30 µM) responses to THC (5 µM) remained unaffected. Administration of O-1918 alone had no influences. (**c**) Impact of GPR55 antagonist CID16020046 (CID) on THC effects. The Ki67-reducing effects of THC (5 µM) were effectively attenuated when CID (10 µM) was co-applied, whereas CID alone induced no alterations in the number of Ki67-immunoreactive nuclei in comparison to the untreated control group. To block each receptor, we applied the corresponding antagonist 15 min (++) before THC was subsequently added. Data are means ± SEM of *N* = 3 independent experiments performed in duplicate. Significance was chosen for *p* < 0.05. The asterisk denotes significant results regarding the respective measurement indicated with the bar.

We next studied the role of CB_1_ and CB_2_ in the THC-mediated suppression of Ki67^+^ cells by blocking the receptors with their specific antagonists AM281 (CB_1_) and AM630 (CB_2_), respectively. Cells were pre-incubated with each antagonist for 15 min (++) and then treated with THC for 24 h. After pre-treatment with AM281 and AM630, the responses to THC remained unaffected, making a participation of CB_1_ and CB_2_ in the observed effects very unlikely (Figure 4a, Table S3). Cells treated with AM281 or AM630 alone or a combination of both showed no alterations in the number of Ki67^+^ nuclei when compared to control cells (Figure 4a and Figure S2, Table S3).

Furthermore, the highly selective CB_1_ agonist ACEA (10 µM) and CB_2_ agonist JWH133 (10 µM) were ineffective to influence the cell cycle of *GBM #4* (Figure S2a,d). In contrast, both substances reduced Ki67^+^ cells of *GBM #10* after 24 h (Figure S2b,d). In GBM #23, only ACEA attenuated the number of Ki67^+^ cells significantly (Figure S2c,d). In contrast to the THC-mediated effects, effects triggered by ACEA or JWH133 were fully abolished after pretreatment with the respective receptor antagonists (Figure S2b,d), indicating an involvement of CB_1_- and CB_2_-dependent mechanisms in *GBM #10* and *GBM #23*.

Given the orphan cannabinoid receptor GPR18 also being a target of THC [19,28], we used O-1918 as an antagonist for GPR18. Our data revealed that inhibition of GPR18 by O-1918 did not abolish THC effects (Figure 4b, Table S3), suggesting a GPR18-independent mechanism for THC-mediated Ki67 reduction.

### 2.5. GPR55 Activation Was Involved in THC-Dependent Reduction of Ki67^+^ Cells

CBD was previously shown to antagonize GPR55 activation and to counteract THC effects [25,29]. Therefore, we checked whether THC effects were mediated via GPR55. We found that pharmacological blockade of GPR55 by 10 µM CID, a synthetic GPR55 antagonist, was sufficient to inhibit the suppression of Ki67^+^ cells by THC in *GBM #4* and *GBM #10* (Figure 4c and Figure S3a–c, Table S3), suggesting GPR55-dependent signaling. CID alone had no statistically significant effect on Ki67^+^ cells, neither in *GBM #4* nor in *GBM #10* (Figure 4c, Table S3) and *GBM#23* (Figure S3a,d). Notably, a co-stimulation of THC with CID also did not cause any alterations of Ki67^+^ cells in *GBM #23* (Figure S4a,d).

**Figure 5:**
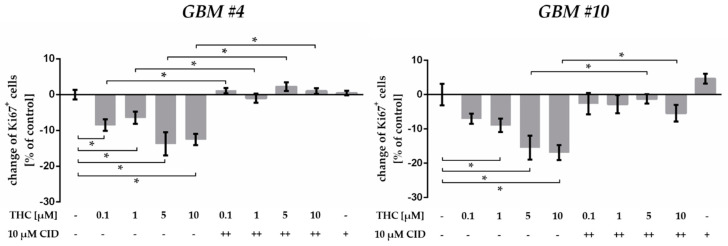
Effects of increasing THC concentrations in presence of GPR55 antagonist CID16020046 (CID). In comparison to the untreated control group, application of different THC concentrations (0.1–10 µM) for 24 h led to a significant reduction in the number of Ki67^+^ cells in *GBM #4* and *GBM #10*. The Ki67-reducing effects of THC were effectively attenuated when CID (10 µM) was co-applied, whereas CID alone induced no alterations. To block the GPR55 receptor, we applied CID 15 min (++) before THC was subsequently added. Data are means ± SEM of *N* = 3 independent experiments performed in duplicate. Significance was chosen for *p* < 0.05. The asterisk denotes significant results regarding the respective measurement indicated with the bar.

As reported earlier, biphasic effects of cannabinoids might be observed after using different concentrations applied to the same cell line [30]. Therefore, *GBM #4* and *GBM #10* cells were treated with 0.1 µM, 1 µM, and 10 µM THC in addition to 5 µM (Figure 5, Table S4). The number of Ki67^+^ cells was significantly reduced at all concentrations, with smaller effects for 0.1 µM or 1 µM and more pronounced effects for 5 µM or 10 µM THC (Figure 5, Table S4). The THC effects were also effectively abolished in presence of GPR55-antagonist CID (10 µM) (Figure 5, Table S4).

**Figure 6:**
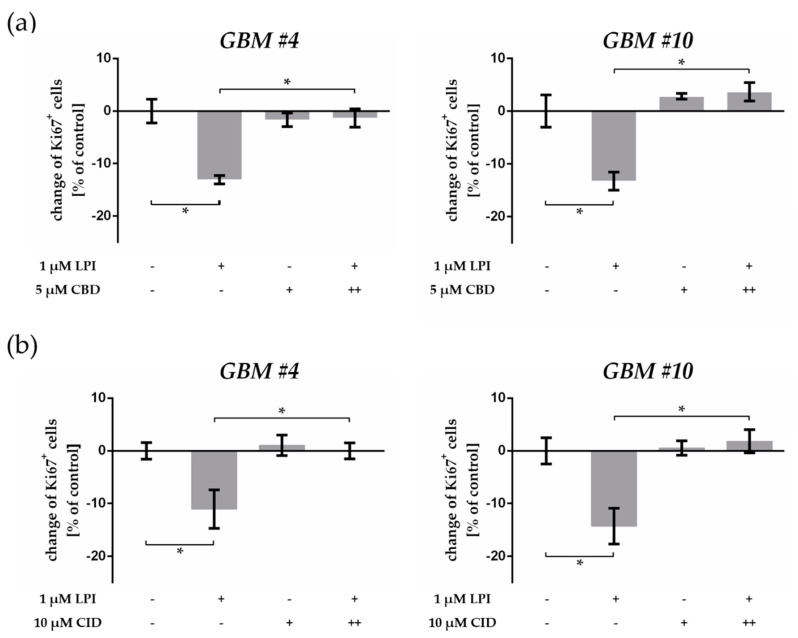
Effects of the endogenous GPR55 ligand lysophosphatidylinositol (LPI). (**a**) Impact of LPI on the number of Ki67-labelled GBM cells and impact of CBD on LPI effects. Application of LPI (1 µM) led to a decrease of Ki67^+^ cells in *GBM #4* and *GBM #10* compared to the control group. As obtained for THC, LPI effects were significantly prevented by a combination with CBD (5 µM), whereas CBD alone had no effects. (**b**) Impact of GPR55 antagonist CID16020046 (CID) on LPI effects. In the presence of GPR55 antagonist, CID (10 µM) responses to LPI were effectively inhibited. Cells treated with CID alone were unaffected compared to the control group. To block GPR55, CBD and CID were applied 15 min (++) before LPI was subsequently added. Data are means ± SEM of *N* = 3 independent experiments performed in duplicate. Significance was chosen for *p* < 0.05. The asterisk denotes significant results regarding the respective measurement indicated with the bar.

To confirm the involvement of GPR55, we investigated the effect of LPI, an endogenous agonist of GPR55 [22]. In similarity to data obtained by THC, LPI significantly decreased the percentage of Ki67^+^ nuclei in both *GBM #4* and *GBM #10* (Figure 6a, Table S5).

Thereafter, the cells were pre-incubated with CBD or CID before LPI was subsequently added. Either CBD or CID diminished LPI-mediated Ki67 reduction (Figure 6a,b, Table S5), confirming the binding of LPI to GPR55 and consequently revealing the binding of THC to GPR55 as well as the putative antagonist activity of CBD at GPR55. Although GPR55 was detected in *GBM #23*, stimulation with LPI and CBD or CID alone and in combination did not influence the number of Ki67-immunoreactive nuclei in these cells (Figure S4b–d).

**Figure 7:**
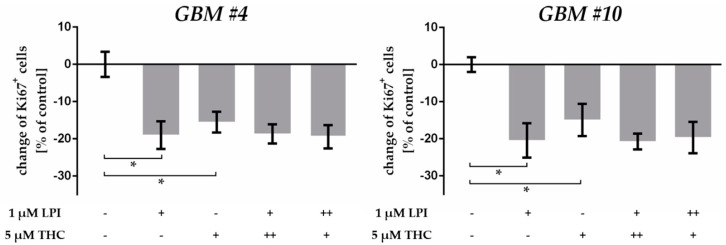
Co-stimulation of THC and LPI. In comparison to THC- and LPI-treated groups, co-application of LPI (1 µM) and THC (5 µM) caused no additive effects, regardless of the order of 15 min pre-treatment (++) with THC or LPI. Data are means ± SEM of *N* = 3 independent experiments performed in duplicate. Significance was chosen for *p* < 0.05. The asterisk denotes significant results regarding the respective measurement indicated with the bar.

To analyze whether additive effects can be achieved, we incubated the cells with a combination of THC and LPI. As aforementioned, LPI and THC alone decreased the number of Ki67^+^ cells in *GBM #4* and *GBM #10* significantly, but caused no additive effects after co-administration, indicating that both substances might bind to the same receptor site (Figure 7, Table S6). Additionally, missing additive effects were independent of the order of 15 min pre-treatment (++) with THC or LPI (Figure 7, Table S6).

## 3. Discussion

Among the extremely pronounced heterogeneity, uncontrolled cell growth is an additional hallmark for the aggressive phenotype of GBM [3]. Both parameters represent an important aspect accounting in large part for the poor prognosis of GBM, highlighting the urgency of developing alternative therapy modalities and thereby improving patient care and clinical outcomes. Evidence has accumulated that phytocannabinoids, especially THC and CBD, can be beneficial for treatment of tumor diseases directly as anti-tumor agents by preventing tumor-associated mechanisms such as proliferation, migration, invasion, and angiogenesis [9,10,11,14,31,32]. These effects of THC and CBD were conducted by a variety of cannabinoid-specific receptors. Therefore, it is needed to evaluate the responsible receptor behind specific cannabinoid-mediated effects in individual tumor cells. Thus, the present study focused on the identification of the receptor involved in the modulation of the number of Ki67^+^ cells from patient-derived GBM cells after THC exposure by investigating the impact of different receptor-specific agonists and antagonists on THC acting.

### 3.1. THC Modified the Percentage of Ki67^+^ Patient-Derived GBM Cells

In this study, we analyzed the impact of THC and CBD on the proliferation capacity of human patient-derived GBM cells, measured by counting the number of Ki67-immunoreactive cells. A recent meta-analysis confirmed that in histological sections Ki67 is a sensible indicator of poor prognosis in glioma patients [33]. Previous studies reported on Ki67 indices of GBM tumor samples ranging from 0% to 76.4% [34,35,36]. In our study, high growth fractions between 69% and 85% were detected, indicating an uncontrolled growth of these GBM cells. The higher numbers of Ki67^+^ cells in our study compared to the in vivo situation might be explained by the fact that only those tumor cells with strong proliferation capacity were permanently preserved in cell culture, and other tumor-associated cell types having low proliferation rates, such as immune cells and endothelial cells, are usually lost due to the culture condition.

However, we observed a significant decrease of Ki67^+^ fractions in two of the three GBM cells after stimulation with THC, while CBD did not cause any alterations. A decrease in Ki67^+^ cells implies an accumulation of senescent and non-divisible cells (G_0_-phase) and indicates a THC-induced cell cycle arrest. Indeed, using FACS analysis of U251-MG GBM cells, THC was earlier reported to induce cell cycle arrest in the G_0_-G_1_ phase, but not in the G_2_-M or S [14]. In addition, investigations in breast cancer cell line EVSA-T and in melanoma cell line A375 showed an increased number of cells in the G_0_-G_1_ compartment and, in parallel, a decreased number of cells in S phase after THC treatment [10,32]. Modulation of essential regulators of the mammalian cell cycle such as downregulation of cdc2, inhibition of AKT, or hypophosphorylation of pRb retinoblastoma protein tumor suppressor were described as the underlying mechanism for cannabinoid-induced cell cycle arrest [10,32]. Furthermore, evidence exists for the cell cycle dependent induction of apoptosis, leading subsequently to an accumulation of cells in certain cell cycle phases. FACS analysis of THC-treated EVSA-T cells revealed that THC-induced cell cycle arrest was associated with apoptosis in all phases, but the majority of apoptotic cells were detectable in the G_2_-M compartment [10]. In our *GBM #4* and *GBM #10* cells, one might assume a THC-mediated accumulation of cells in the G_0_-G_1_ phase paralleled by a specific reduction of cells in the S or G_2_-M phase by apoptosis. In further investigations, the evaluation of the functional meaning of an altered number of Ki67^+^ cells after THC treatment should be performed, and its relevance for anti-tumor actions of THC clarified.

Interestingly, an enhancement of THC-induced cell cycle arrest and apoptosis was measured by flow cytometry in U251-MG cells when THC and CBD were co-applied [12,14]. In that study, blocking the CB_1_ or CB_2_ receptors abrogated THC- but not CBD-induced effects, indicating a CB_1_-/CB_2_-independent acting of CBD. Therefore, activation of different intracellular mechanisms via different receptors was assumed for THC and CBD to promote cell cycle arrest and apoptosis [14]. In contrast to these reports, THC and CBD exhibited opposed effects in the present work, as CBD antagonized the anti-Ki67 effect of THC. This interference might be explained either by cell type-specific differences or by binding of both substances to the same receptor.

### 3.2. THC Effects Were Not Mediated via CB_1_ and CB_2_

In human GBM cell lines, the ability of THC to inhibit growth and induce apoptosis has been linked to the activation of CB_1_ and CB_2_ receptors, which was verified by using selective receptor antagonists or by silencing receptor expression with specific small interfering RNAs (siRNAs) [12,14]. We used AM281 and AM630, both selective antagonists of CB_1_ and CB_2_, to underline the involvement of the two classical cannabinoid receptors. Notably, CB_1_ and CB_2_ were detectable in all GBM cells tested, and incubation with synthetic agonists ACEA (CB_1_) and JWH133 (CB_2_) caused a reduction of Ki67^+^ cells in *GBM #10* and *GBM #23*, but not in *GBM #4*. These effects were prevented in the presence of AM281 or AM630, confirming their CB_1_- and CB_2_-specfic interactions. In contrast, AM281 and AM630 were unable to diminish the altered percentage of Ki67^+^ cells upon exposure of THC in *GBM #4* and *GBM #10*. Although THC is described as a potent CB_1_ and CB_2_ ligand with similar K_i_-values to CB_1_ (K_i_ = 5.05 nM) and CB_2_ (K_i_ = 3.13 nM) compared to ACEA (K_i_ = 1.4 nM) and JWH133 (K_i_ = 3.4 nM) [17,37,38], our data indicate a CB_1_/CB_2_-independent signaling of THC.

One possible explanation for the reason why THC did not act in a similar way as ACEA and JWH133 might be attributed to the biased signaling of cannabinoid receptors (functional selectivity). Functional selectivity occurs when one effector becomes preferentially activated by a ligand more potently and efficaciously than another through ligand-specific changes in receptor conformations [39]. Several studies demonstrated that the ability of THC to stimulate different effectors (G-proteins and β-arrestin) differs from agonists for CB_1_ and CB_2_ receptors [40,41,42]. In general, CB_1_ and CB_2_ are mainly linked to Gα_i/o_ type G-proteins, but under special circumstances CB_1_ can also recruit other G-protein types such as Gα_s_ and Gα_q_ or other proteins for signaling such as β-arrestin [43,44,45]. In an in vitro analysis of neurons with wild-type or Huntington disease background, THC was signal biased towards β-arrestin, Gα_q_, and Gβγ compared to Gα_i/o_, whereas AEA was signal biased towards Gα_i/o_ compared to β-arrestin and Gα_q_ [41]. In line with that study, a maximal Gα_i_ activation via CB_1_ was reported for the non-specific agonists HU-210 and AEA, whereas only a partial Gα_i_ activation was detected for THC [40]. In addition, in murine brain cortex samples, Gα_i_ subfamily member Gα_i3_ was triggered by ACEA, but not by THC [42]. This is in a good agreement with our observation in *GBM #23*, where ACEA reduced Ki67^+^ cells CB_1_-dependently, but THC did not exhibit any effects.

Consequently, these reports suggest that in our experiments, THC, ACEA, and JWH133 might induce distinct active receptor conformations of CB_1_ and CB_2_, which in turn might favor different effectors. The results might be the transduction of different signaling pathways through the same receptor. As observed here, only ACEA- and JWH133-meditated signaling pathways were able to reduce Ki67^+^ cells via CB_1_ or CB_2_.

### 3.3. THC Did Not Act via GPR18

In addition to CB_1_ and CB_2_, THC can also bind to the orphan cannabinoid receptor GPR18 as a potent agonist when ectopically expressed in HEK293 cells [28]. GPR18 is highly abundant in testes, spleen, and within the brain, and its mRNA was evident in detectable amounts in cell lines of a few tumor entities, including melanoma, breast cancer, and GBM [46,47,48]. However, the precise role of GPR18 in tumor diseases remains unclear. GPR18 is a Gα_i/o_-coupled receptor and it seems conceivable that its activation leads to similar effects as reported for the activation of CB_1_ or CB_2_. However, no altered phosphorylation of extracellular signal-regulated kinase1/2 (ERK1/2) was found after stimulation of two primary GBM cells (NZB11, NZB19) with N-arachidonoyl glycine, the endogenous GPR18-agonist [48]. Because of the GPR18 expression in the here-examined patient-derived GBM cells, we speculated this receptor might be responsible for anti-Ki67 activities of THC. Antagonization of GPR18 by its selective antagonist O-1918 showed no effects on cell cycle alone and after THC treatment. Consequently, GPR18 was ruled out for modifying Ki67 upon stimulation with THC.

### 3.4. THC and CBD Exhibited GPR55-Dependent Signaling in Patient-Derived GBM Cells

A thigh relationship to the endocannabinoid system was demonstrated for another orphan GPCR, namely, GPR55 [49]. Its wide distribution within the body led to the assumption that GPR55 might be involved in the regulation of diverse physio-pathological functions including endocrine function, tissue inflammation, and energy metabolism [50,51,52]. More and more studies have been conducted to address the relevance of GPR55 in the context of human tumor diseases [23,53]. Recently, the GPR55 was demonstrated as having an association with tumor cell proliferation of different tumor cell lines. A downregulation of GPR55 by specific siRNAs blocked proliferation of EVSA-T (breast), T98G (GBM), MIA PaCA-2 (pancreatic), OVCAR3 (ovarian), and PC3 (prostate) cells [23,25]. In addition, the ability of GPR55 to promote tumor cell proliferation was also reported in vivo, where a receptor knockdown diminished tumor growth in a xenograft-based model of GBM [23]. Although the specific pharmacology of GPR55 remains controversial, most evidence agrees on its activation by LPI [22]. It was postulated that the LPI/GPR55 axis plays a crucial role in tumor progression, and that blocking this pathway might be a promising strategy to inhibit cancer-promoting mechanisms, especially tumor cell proliferation. In our study, GPR55 was detectable in patient-derived tumor cells of human GBMs. We asked whether GPR55 might mediate the CB_1_/CB_2_-independent effects of THC in our system. The status of GPR55 as a putative cannabinoid receptor is still controversial. It is well accepted that GPR55 is not only activated by LPI but also can recognize certain CB_1_ and CB_2_ receptor ligands [54]. Nevertheless, it should be considered that most of pharmacological analyses have investigated the effects of cannabinoids on GPR55 in cell lines ectopically overexpressing GPR55 [55]. GPR55 was reported to bind THC as an agonist and CBD as an antagonist [29]. In HEK293 cells transiently expressing human GPR55 and in neurons of dorsal root ganglion, THC (5 µM) evoked an intracellular calcium increase via GPR55 activation [56]. Furthermore, CBD was able to block GPR55-dependent signaling of LPI in prostate and ovarian carcinoma cells [25]. In this work, CBD exhibited an antagonistic activity by counteracting THC effects without showing any effects on its own. These results led to the hypothesis of the involvement of GPR55 in THC actions. By using the selective GPR55 antagonist CID, we demonstrated that THC indeed interacts with GPR55, causes an activation of the receptor, and modulates the Ki67^+^ fractions of patient-derived GBM cells. Additionally, the results confirm the antagonistic effect of CBD. Furthermore, it is known that cannabinoids display biphasic effects, which might occur when they were used in different concentrations in the same cell line [30]. At concentrations ≤1 µM, THC acted as a CB_2_ agonist, and at concentrations ≥5 µM, THC acted as an antagonist via cross antagonism by GPR55 through a CB_2_-GPR55 heteromer [30]. Thus, we treated the cells exemplarily with concentrations ranging between 0.1 and 10 µM THC. Comparable effects, but in different intensities, were observed, which were prevented by CID. It seems that biphasic effects did not occur regarding the modulation of Ki67 in the investigated cells, and here THC acts only via GPR55.

**Figure 8:**
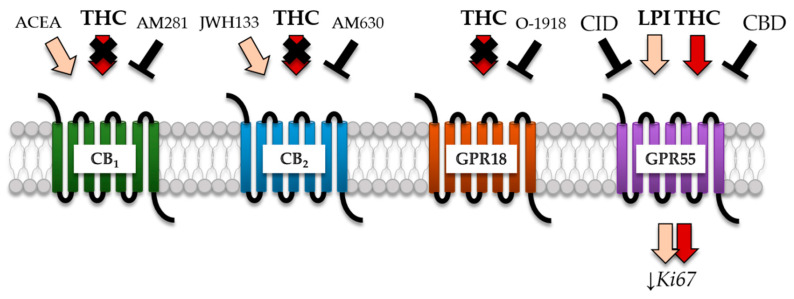
Proposed model for action of THC to modify Ki67 fractions in the considered GBM cells. GPR55 was identified as a potential receptor for the anti-Ki67 action of THC in human patient-derived GBM cells by systematically investigating the impact of different receptor-specific antagonists on THC acting. Despite the fact that AM281 (CB_1_-antagonist) and AM630 (CB_2_-antagonist) diminished the effects of ACEA (CB_1_-agonist) and JWH133 (CB_2_-agonist), both were not able to inhibit THC, indicating a CB_1_/CB_2_-independent mechanism. Furthermore, THC effect remained unaffected after application of O-1918 (GPR18-antagonist). Finally, CID and CBD are described as antagonists for GPR55 and indeed exerted antagonist activity in presence of THC, suggesting a GPR55-dependent signaling. Similar data were obtained after the stimulation with LPI (GPR55-agonist), confirming the participation of GPR55 in this process.

The observation of similar findings upon treatment with GPR55 agonist LPI and attenuation of its effects by CBD and CID strongly support the conclusion that the decreased Ki67^+^ fractions detected in *GBM #4* and *GBM #10* upon THC treatment were mainly driven by the activation of GPR55 (Figure 8).

Notably, our results are in contrast to the aforementioned proliferation-promoting effects of GPR55 in different tumor entities, since an activation of GPR55 by LPI and even by THC led to a decreased Ki67-immunoreactive nuclei of GBM cells, which implies a growth-inhibiting effect of both substances. Furthermore, GPR55 antagonists CID and CBD did not affect proliferation of GBM cells after 24 h, although an inhibition of tumor cell proliferation of the pancreatic cell line PANC1 with both GPR55 antagonists was already reported [57]. Similar contradictory results were obtained by a previous study, demonstrating a growth inhibition of cholangiocarcinoma cell line Mz-ChA-1 when GPR55 became activated by AEA, an endocannabinoid that generally acts as an agonist at CB_1_ or CB_2_ [26]. The effect of AEA was still present after treatment with CB_1_ and CB_2_ antagonists or with the specific Gα_i/o_ inhibitor pertussis-toxin (PTX), but was fully blocked after a stable shRNA-mediated knockdown of GPR55 [26]. In this context, distinct signaling pathways activated by GPR55 were discussed and should be considered in further investigations.

### 3.5. Cell Type-Specific Effects of THC and LPI

Although GPR55 was detected in *GBM #23*, THC and LPI were incapable of modulating the percentage of Ki67-immunoreactive cells. These results suggest that the efficacy of THC in reducing the number of Ki67^+^ cells shows differences between the three considered tumor cells. The distinct genetic background due to the inter- and intratumoral heterogeneity might be responsible for a tumor- and cell-type specific response after activation of GPR55.

Since GBMs are characterized by a very high number of genetic alterations causing changes in proliferation, migration, and invasiveness of tumor cells [58], it is very likely that one downstream effector molecule of GPR55 might be mutated in *GBM #23*, resulting in a disruption or a shift of its signaling. However, the mutation status of effectors involved in GPR55 signaling in the examined patient-derived cells is unknown and remains to be determined.

A further aspect that should be considered is the distinct protein–protein interactions such as variations in accessory protein binding and dimerization with other GPCRs. Functional selectivity might offer another argument with THC as an inducer of cell type-specific conformational changes of GPR55 favoring other G-proteins to stimulate G-protein-specific signaling pathways with distinct consequences for cell fate. There is some evidence in the literature underlying the amount of certain G-proteins as crucial parameters for the signaling outcome of one receptor [43,44]. An interesting study demonstrated in HEK293 cells stably expressing rat CB_1_ that in the presence of PTX, CB_1_ coupling shifted from Gα_i/o_ to Gα_q_ when CB_1_ became activated by WIN 55,212-2 [43]. By inhibiting the Gα_i/o_ subunit, PTX attenuates the interactions of G_i/o_ heterotrimeric G-proteins with the receptor, leading to an increase in the ratio of functional Gα_q_ to Gα_i/o_, making a coupling to less-favored Gα_q_ evident [43]. Similar results were obtained later in HEK293 cells transiently expressing human CB_1_, where PTX leads to a shift from Gα_i_- to Gα_s_-mediated signaling after WIN55,212-2 and THC administration [44]. Additionally, due to Gα_i_ exhaustion, this shift was also detectable when CB_1_ receptor expression was increased [44]. Interestingly, this phenomenon was reversed by increasing the expression level of Gα_i_ protein [44]. It is therefore conceivable that in our study the GBM cells might differ in the assembly and expression levels (over- or underexpression) of certain G-proteins or other effector molecules determining the signaling driven by GPR55 activation.

Furthermore, the signaling cascades of GPR55 can also be changed by the cannabinoid receptors CB_1_ and CB_2_ upon activation, since both are capable of physically interaction with GPR55 [30,59,60]. In HEK293 cells co-transfected with CB_1_ and GPR55, the presence of CB_1_ caused a reduction or even an inhibition of GPR55-mediated signaling [60]. Similarly, after dimerization of GPR55 with CB_2_, a decreased signal transduction was observed when GPR55 became activated [30,59]. Importantly, a cross antagonism phenomenon was described in HEK293 cells transfected with CB_2_ and GPR55, suggesting one receptor can be targeted by using the partner receptor antagonist. In particular, the LPI-induced ERK1/2 phosphorylation was prevented by AM630 or SR144528 (CB_2_ antagonists) [30,59], and the HU-308 (CB_2_ agonist) action was blocked by a GPR55-antagonist HBA [30]. In contrast, blocking of CB_2_ with AM630 did not affect GPR55-mediated NFAT (nuclear factor of activated T cells) activation after LPI application [59]. Here, even the interaction with CB_2_ led to a reduced NFAT activation by the LPI/GPR55 axis [59]. However, in the present work, we detected both receptors CB_1_ and CB_2_ in all examined tumor cells. Since blocking CB_1_ and CB_2_ did not affect GPR55-dependent THC signaling in *GBM #4* and *GBM #10*, it counts against a heterodimerization of GPR55 with CB_1_ and CB_2_ in *GBM #4* and *GBM #10*, as suggested by Moreno and colleagues [30], but presumably explains the opposing results obtained in *GBM #23*.

Nevertheless, the presented data reflect the high heterogeneity of GBMs regarding the sensitivity to cannabinoids and their receptor-dependent signaling pathways. Consequently, if cannabinoids are considered as additional therapeutic agents, their efficacy has to be evaluated in each patient.

## 4. Materials and Methods

### 4.1. Isolation of Human Patient-Derived GBM Cells and Cell Culture

The study was conducted in accordance with the Declaration of Helsinki and was approved by the local ethics committee of the University Halle-Wittenberg (project reference number: 2015-144). All patients provided signed written informed consent.

Primary cultures of brain tumor cells (designated as *GBM #4*, *GBM #10*, and *GBM #23*) were obtained from human biopsies. Some details of the tumor samples from whom the cells were derived and of the isolated cells are shown in Table S1.

To isolate patient-derived GBM cells, we selected a ≈1 mm^3^ volume of the primary tumor material, which we rinsed 3 times with 4 °C cold HBSS (Hank’s balanced salt solution, without Ca^2+^ and Mg^2+^, Invitrogen, Schwerte, Germany, 14170-138) and then incubated with 1 mL mixture of trypsin (4 mg/mL, Invitrogen, Schwerte, Germany, 15090-046) and DNase (0.5 mg/mL, Worthington Biochemical, Lakewood, NJ, USA) on ice for 4 min. After addition of HBSS (4 °C, without Ca^2+^ and Mg^2+^), the supernatant was removed and the pellet was carefully mixed with 100 μL DNase, followed by a washing step by addition of HBSS (4 °C, with Ca^2+^ and Mg^2+^, Invitrogen, Schwerte, Germany, 24020-133) and a centrifugation step (10 min, 4 °C, 800 g). Subsequently, the pellet was suspended in culture medium, transferred to a cell culture flask, and cultured in a humidified incubator at 37 °C and 5% (*v*/*v*) CO_2_. The obtained cells were grown in high-glucose Dulbecco’s modified Eagle’s medium (Invitrogen, Schwerte, Germany, 41965-062) supplemented with 10% (*v*/*v*) fetal bovine serum (FBS; Invitrogen, Schwerte, Germany, 10500-064) and 1% (*v*/*v*) penicillin–streptomycin (Invitrogen, Schwerte, Germany, 15140-122).

### 4.2. Treatment

For cannabinoid treatment, 10,000 cells were seeded on sterile coverslips and placed into 24-well plates (Greiner Bio-One, Frickenhausen, Germany, 662160). Cells were allowed to adhere overnight and were treated for 24 h with fresh medium containing cannabinoids or LPI (Table 1) until they were fixed with 4% (*w*/*v*) paraformaldehyde (AppliChem GmbH, Darmstadt, Germany, 1.414.511.211). For receptor blockades, cells were first pre-incubated with the corresponding antagonists (Table 1) alone for 15 min until the medium was replaced by fresh medium containing agonists with or without antagonists for 24 h. All treatments were carried out in DMEM with 10% (*v*/*v*) FBS.

### 4.3. PCR

Total RNA from cells was extracted using peqGOLD Trifast (Peqlab, Erlangen, Germany, 30-2010) and treated with a DNA-free Kit (Invitrogen, Schwerte, Germany, AM1906), according to the manufacturer’s instructions. RNA concentration was quantified using Synergy Mx Mircroplate Reader (BioTek, Winooski, VT, USA). The generated cDNA (Reverse Transcription System, Promega GmbH, Walldorf, Germany, A3500) was amplified in a total volume of 20 µL containing 10 µL PCR-MasterMix (Promega Inc., M7505), 0.5 µL forward and 0.5 µL reverse primer (25 pM), 0.25 µL of EvaGreen dye (Biotium, Hayward, CA, USA, 31000), 4.75 µL of nuclease-free water (Promega GmbH, Walldorf, Germany, P1193), and 4 µL of cDNA. The PCR reaction was performed in a rotor-cycler (Rotor-Gene RG 6000; Corbett Research, Pty Ltd., Sydney, Australia) under the following conditions: initial denaturation at 95 °C, followed by 40 cycles of denaturation at 94 °C (3 s), annealing at 60 °C, elongation at 72 °C (30 s), and fluorescence detection at 76 °C (*CNR1, CD44, MSI1, NES, SOX2*) or 80 °C (*CNR2, GPR18, GPR55, POLRR2A*) (15 s). PCR products were placed on a 1.5% (*w*/*v*) agarose gel (PeqLab, Erlangen, Germany, 35-1020) containing GelRed (Biotium, Hayward, CA, USA 41003) and were visualized using BioTek Synergy Mix (Biotek, Winooski, VT, USA). The primers used are listed in Table 2.

### 4.4. Western Blot

After protein isolation, the concentration was measured (Pierce BCA Protein Assay Kit; Thermo Scientific, Schwerte, Germany, 23225) and 20 µg of total protein was used for the electrophoresis on 12.5% (*w*/*v*) SDS gels. Gels were transferred onto nitrocellulose membranes (BA 85 6E Protean; Whatman plc/G E Healthcare Life Sciences, Darmstadt, Germany), blocked with Roti-Block (Carl Roth GmbH, Karlsruhe, Germany, A151.2) for 30 min, and incubated overnight at 4 °C with the primary antibody (Table 3) diluted in Roti-Block. Membranes were washed 3 times with washing buffer, incubated with horseradish peroxidase (HRP)-conjugated secondary antibody (Table 3) and washed 5 times. The signal detection was performed using Luminata Classico Western HRP Substrate (Merck, Darmstadt, Germany, WBLUCO0500) and was visualized with FusionFX7. β-Actin was used as a reference.

### 4.5. Immunochemical Staining

Fixed cells were treated with 3% (*v*/*v*) H_2_O_2_ (Carl Roth GmbH, Karlsruhe, Germany, 8070.2) in methanol (Carl Roth GmbH, Karlsruhe, Germany, AE01.2) for 10 min to block endogenous peroxidases and washed 3 times with 0.02 M PBS containing 0.3% (*v*/*v*) Triton X-100 (AppliChem GmbH, Darmstadt, Germany, A1388.0500) (PBS/Triton). Unspecific binding sides were blocked with normal goat serum (1:20, Jackson ImmunoResearch, West Grove, PA, USA, 005-000-121). The samples were incubated overnight with primary antibody (Table 3) diluted in PBS containing 0.5% (*w*/*v*) bovine serum albumin (Sigma-Aldrich^®^ Chemie GmbH, Steinheim, Germany, A7906) and 0.3% (*v*/*v*) Triton X-100. After washing with PBS/Triton, samples were incubated with rabbit-specific biotinylated secondary antibody (Table 3) followed by ExtrAvidin peroxidase (Sigma-Aldrich® Chemie GmbH, Steinheim, Germany, E2886). Staining was developed with 3,3′-diaminobenzidine (DAB; Sigma-Aldrich^®^ Chemie GmbH, Steinheim, Germany, D8001) in the presence of 0.05% (*v*/*v*) H_2_O_2_ for 5 min. Finally, the cells were counterstained using Mayer’s hematoxylin (Carl Roth GmbH, Karlsruhe, Germany, T865.1), dehydrated in an ascending concentration gradient of ethanol, cleared with xylene (Carl Roth GmbH, Karlsruhe, Germany, 97.13.3), and covered with Entellan (Merck, Darmstadt, Germany, 107960).

All images were obtained with 20× objective with a Leica DMi8 microscope (Wetzlar, Germany). For Ki67 analysis, 5 different, random regions per coverslip were recorded and analyzed. Ki67 is expressed during the whole cell cycle, except for G_0_ (Figure 1b). The population of Ki67-positive (Ki67^+^) cells represents the cell fraction, which is able to proliferate (growth fraction). Thus, the number of Ki67-labelled and hematoxylin-positive cells was counted and the percentage of Ki67^+^ cells calculated.

### 4.6. Statistics

The values were presented as a mean with standard error of the mean, and all groups were normalized to the untreated control group. Statistics were calculated using Student’s *t*-test and one-way ANOVA with Tukey’s post-test using GraphPad Prism6. All *p*-values refer to the respective untreated group of the same parameter of the same cells or to the group treated with the agonist for the examined receptor. Significance was chosen for *p* < 0.05.

## 5. Conclusions

In this study, we added further insights into THC anti-tumor action, showing its ability to modify the number of Ki67^+^ cells of human patient-derived GBM cells, particularly through the activation of the orphan receptor GPR55 without affecting CB_1_ and CB_2_ receptors as usual targets. Although GPR55 was detected in all examined cells, application of THC affected the Ki67 positivity only in two of them. The understanding of the signaling pathways triggered by GPR55 will help to elucidate the cell type-specific differences and additionally the somewhat conflicting results from the present study to the literature. Nevertheless, THC seems to be a promising compound for individual treatment of those GBMs with functional GPR55 receptors.

## Figures and Tables

**Table 1:** Cannabinoids and LPI.

Substances	Targets	Behavior	Solvent	Concentration	Company	Article Number
ACEA	CB_1_	agonist [61]	ethanol	10 µM [11,15,16]	Tocris, Bristol, UK	1319
	SMILES: CCCCC/C=C\C/C=C\C/C=C\C/C=C\CCCC(=O)NCCCl					
AM281	CB_1_	antagonist [62]	DMSO	1 µM [15,16]	Tocris, Bristol, UK	1115
	SMILES: CC1=C(N(N=C1C(=O)NN2CCOCC2)C3=C(C=C(C=C3)Cl)Cl)C4=CC=C(C=C4)I					
AM630	CB_2_	antagonist [63]	DMSO	1 µM [15,16]	Tocris, Bristol, UK	1120
	SMILES: CC1=C(C2=C(N1CCN3CCOCC3)C=C(C=C2)I)C(=O)C4=CC=C(C=C4)OC					
Cannabidiol (CBD)	CB_1_ CB_2_	weak agonist [19]	DMSO	5 µM [64]	Tocris, Bristol, UK	1570
	GPR18 GPR55	antagonist [19]				
	SMILES: CCCCCC1=CC(=C(C(=C1)O)C2C=C(CCC2C(=C)C)C)O					
CID16020046	GPR55	antagonist [65]	DMSO	10 µM (Figure S2)	Tocris, Bristol, UK	4959
	SMILES: CC1=CC=C(C=C1)C2=NNC3=C2C(N(C3=O)C4=CC=C(C=C4)C(=O)O)C5=CC(=CC=C5)O					
Dronabinol (THC)	CB_1_ CB_2_ GPR18 GPR55	agonist [19]	DMSO	5 µM [64]	THC pharm GmbH, Frankfurt am Main, Germany	THC-1016
	SMILES: CCCCCC1=CC(=C2C3C=C(CCC3C(OC2=C1)(C)C)C)O					
JWH133	CB_2_	agonist [61]	DMSO	10 µM [11,15,16]	Tocris, Bristol, UK	1343
	SMILES: CCCC(C)(C)C1=CC2=C(C=C1)C3CC(=CCC3C(O2)(C)C)C					
Lysophosphatidylinositol (LPI)	GPR55	agonist [22]	DMSO	1 µM [66]	Sigma-Aldrich^®^ Chemie GmbH, Steinheim, Germany	L7635
	SMILES: CC(=O)OCC(COP(=O)(O)OC1C(C(C(C(C1O)O)O)O)O)O					
O-1918	GPR18	antagonist [67]	DMSO	30 µM [68]	Tocris, Bristol, UK	2288
	SMILES: CC1=CC(C(CC1)C(=C)C)C2=C(C=C(C=C2OC)C)OC					

**Table 2:** Primer.

Gene	Accession Number	Forward Primer (5′-> 3′)	Reverse Primer (5′-> 3′)	Product Size
*CD44*	NM_000610	CTGGCGCAGATCGATTTGAA	TTGCTGCACAGATGGAGTTGG	244 bp
*CNR1*	NM_033181	GCATCCAAGGAAGGGATGTA	CCGTTGTGTGTCTCATCCAC	250 bp
*CNR2*	NM_001841	GCTCCTCATCTGTTGGTTCC	TGACCATGGAGTTGATGAGGC	208 bp
*GPR18*	NM_005292	CCACCAAGAAGAGAACCAC	GAAGGGCATAAAGCAGACG	596 bp
*GPR55*	NM_005683	GGTGCTCTCCCTCCCATT	GCTCACCAGTAGCGGGTAAC	172 bp
*MSI1*	NM_002442	GATGGTCACTCGGACGAAGAA	CAAACCCTCTGTGCCTGTTG	149 bp
*NES*	NM_006617	CAGCGTTGGAACAGAGGTTGG	TGGCACAGGTGTCTCAAGGGTAG	389 bp
*POLR2A*	NM_000937	CTTGCCCCGTGCCATGCAGA	CTCGCACCCGGCCTTCCTTG	83 bp
*SOX2*	NM_003106	ACTCCTACGTGGGCGACGAGG	CAGGTCCAGACGCAGGATGGC	389 bp

**Table 3:** Antibodies.

Antibodies	Species	Concentration (Application)	Company	Article Number
Anti-β-actin	mouse	1:5000 (WB ^1^)	Cell Signaling, Danvers, MA, USA	3700
Anti-CB_1_	rabbit	1:200 (ICC ^2^) 1:1200 (WB)	Cayman Chemicals, Ann Arbor, MI, USA	101500
Anti-CB_2_	rabbit	1:200 (ICC, WB)	Alomon Labs, Jerusalem, Israel	ACR-002
Anti-CD133	mouse	1:40 (ICC)	Miltenyi Biotec, Bergisch, Gladbach, Germany	130-092-395
Anti-GPR18	rabbit	1:300 (ICC)	gifted from Ken Mackie	described in [69]
Anti-GPR55	rabbit	1:200 (ICC)	Cayman Chemicals, Ann Arbor, MI, USA	10224
Anti-IDH1 R132H	mouse	1:500 (ICC)	Dianova GmbH	DIA-H09
Anti-Ki67	rabbit	1:200 (ICC)	DSC innovative Diagnostic-System, Hamburg, Germany	KI681C002
Anti-mouse IgG horseradish peroxidase (HRP)-conjugated	horse	1:10,000 (WB)	Vector, Burlingame, CA, USA	PI-2000
Anti-rabbit IgG, biotin conjugated	goat	1:100 (ICC)	Sigma-Aldrich® Chemie GmbH, Steinheim, Germany	B7389
Anti-rabbit IgG, horseradish peroxidase (HRP)-conjugated	goat	1:20,000 (WB)	Vector, Burlingame, CA, USA	PI-1000

^1^ Western blot, ^2^ immunocytochemistry.

## Data Availability

The datasets of the current study are available from the first author on reasonable request.

## Supplementary Materials

The following are available online at https://www.mdpi.com/2072-6694/13/5/1064/s1. Figure S1: Original images of PCR products and Western Blots.; Figure S2: Influence and receptor dependence of CB_1_ and CB_2_ ligands on the percentage of Ki67^+^ cells; Figure S3: Establishment of GPR55 antagonist CID16020046 (CID); Figure S4: Impact of GPR55 ligands on the percentage of Ki67^+^ cells in *GBM #23;* Table S1: Data in patients and primary tumor samples of investigated GBM; Table S2: Exact measurement values and sample sizes after treatment with THC and CBD; Table S3: Exact measurement values and sample sizes after treatment with THC in presence of different antagonists; Table S4: Exact measurement values and sample sizes after treatment with increasing concentrations of THC in presence of CID; Table S5: Exact measurement values and sample sizes after treatment with LPI and LPI in presence of CBD and CID; Table S6: Exact measurement values and sample sizes after treatment with LPI and THC co-application.

## Abbreviations

CB: cannabinoid receptor; CBD: cannabidiol; ECS: endocannabinoid system; GBM: glioblastoma; GPR: G-protein-coupled receptor; LPI: lysophosphatidylinositol; THC: tetrahydrocannabinol.
