# Supplementary material for: THC Reduces Ki67-Immunoreactive Cells Derived from Human Primary Glioblastoma in a GPR55-Dependent Manner
Source: Cancers (Basel). 2021 Mar 3;13(5):1064. doi: 10.3390/cancers13051064 (PMC7959141; doi:10.3390/cancers13051064)
Supplement: Supplementary file 1 [file cancers-13-01064-s001.pdf]

# Supplementary Materials:

# THC Reduces Ki67-Immunoreactive Cells Derived from Human Primary Glioblastoma in a GPR55-Dependent Manner

**Marc Richard Kolbe [1], Tim Hohmann [1], Urszula Hohmann [1], Chalid Ghadban [1], Ken Mackie [2], Christin Zöller [3], Julian Prell [3], Jörg Illert [3], Christian Strauss [3] and Faramarz Dehghani [1,*]**

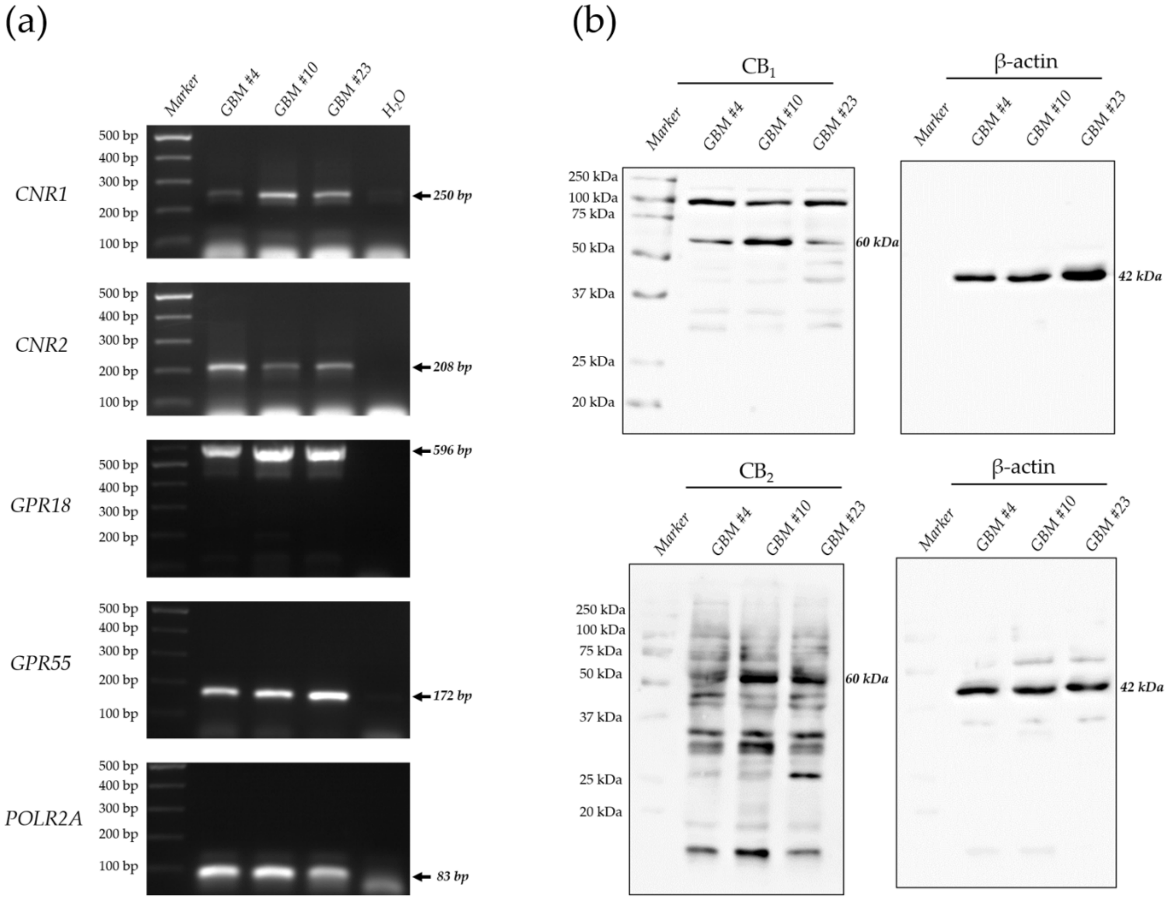


**Figure 1.** Original images of PCR products and Western Blots.

(a)

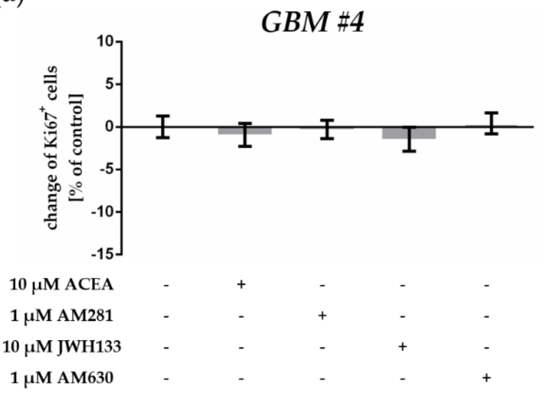


(b)

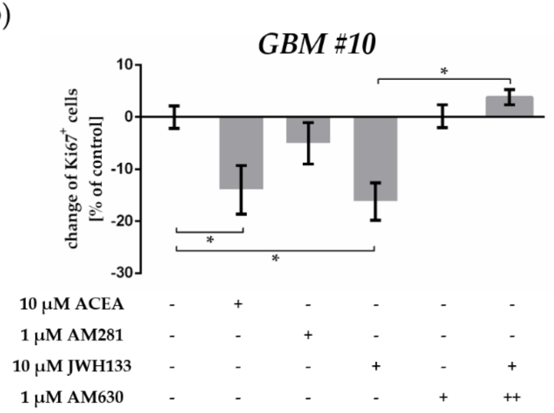


(c)

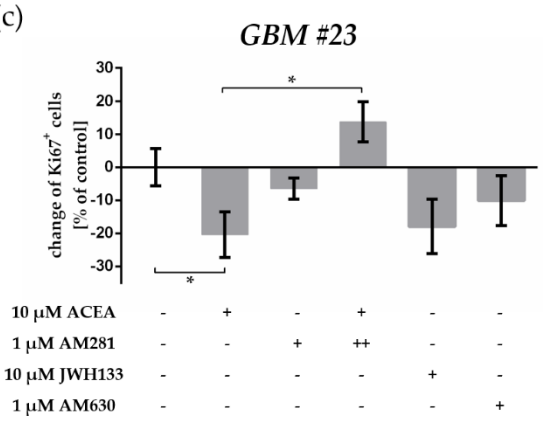


(d)

| *receptor* | *cell type* | *treatment* | *mean [%]* | *SEM [%]* | *sample size* |
|---|---|---|---|---|---|
| | *GBM #4* | CTL | 0.00 | 1.23 | 10 |
| $CB_1$ | *GBM #4* | ACEA | -0.93 | 1.36 | 10 |
| | *GBM #4* | AM281 | -0.31 | 1.08 | 10 |
| $CB_2$ | *GBM #4* | JWH133 | -1.45 | 1.41 | 10 |
| | *GBM #4* | AM630 | 0.41 | 1.22 | 8 |
| | *GBM #10* | CTL | 0.00 | 2.14 | 16 |
| $CB_1$ | *GBM #10* | ACEA | -13.97 | 4.67 | 10 |
| | *GBM #10* | AM281 | -5.02 | 3.99 | 10 |
| | *GBM #10* | JWH133 | -16.24 | 3.58 | 16 |
| $CB_2$ | *GBM #10* | AM630 | 0.16 | 2.18 | 16 |
| | *GBM #10* | JWH133 + AM630 | 3.81 | 1.46 | 6 |
| | *GBM #23* | CTL | 0.00 | 5.65 | 8 |
| | *GBM #23* | ACEA | -20.35 | 6.91 | 8 |
| $CB_1$ | *GBM #23* | AM281 | -6.46 | 3.15 | 8 |
| | *GBM #23* | ACEA + AM281 | 13.73 | 6.05 | 3 |
| $CB_2$ | *GBM #23* | JWH133 | -17.9 | 8.24 | 5 |
| | *GBM #23* | AM630 | -10.07 | 7.60 | 5 |

**Figure S2:** Influence and receptor dependence of $CB_1$ and $CB_2$ ligands on the percentage of $Ki67^+$ cells. The cannabinoids ACEA ($CB_1$-agonist, 10 μM), JWH133 ($CB_2$-Agonist, 10 μM), AM281 ($CB_1$-antagonist, 1 μM) and AM630 ($CB_2$-antagonist, 1 μM) were used to target $CB_1$ and $CB_2$ receptor in *GBM #4*, *GBM #10* and *GBM #23*. **(a)** Impact of $CB_1$- and $CB_2$-ligands on the number of $Ki67^+$ cells of *GBM #4*. Cells remained unaffected after treatment with ACEA, JWH133, AM281 and AM630 for 24 h. **(b)** Impact of $CB_1$- and $CB_2$-ligands on number of $Ki67^+$ cells of *GBM #10*. Application of ACEA and JWH133 led to a significant decreased number of $Ki67^+$ cells compared to the control group. AM281 and AM630 alone caused no changes. By co-application with AM630, the effect of JWH133 was significantly abolished and $CB_2$-activation by JWH133 was identified as underlying mechanism. **(c)** Impact of $CB_1$- and $CB_2$-ligands on number of Ki67-immunoreactive cells of *GBM #23*. Treatment with different $CB_1$ and $CB_2$ ligands revealed a reduced number of Ki67-labelled cells upon ACEA exposure in comparison to the control group. This process was dependent on the activation of $CB_1$, since $CB_1$-antagonist AM281 prevented ACEA-mediated effect when both were co-applied. JWH133, AM281 and AM630 alone caused no significant changes. **(d)** Exact measurement values and sample sizes after treatment with $CB_1$- and $CB_2$-ligands. To completely block $CB_1$ and $CB_2$, antagonists were applied 15 min (++) before agonists were subsequently added. Data are means ± SEM of N=3-8 independent experiments performed in duplicate. Significance was chosen for $p<0.05$. The asterisk denotes significant results regarding the respective measurement indicated with the bar.

(a)

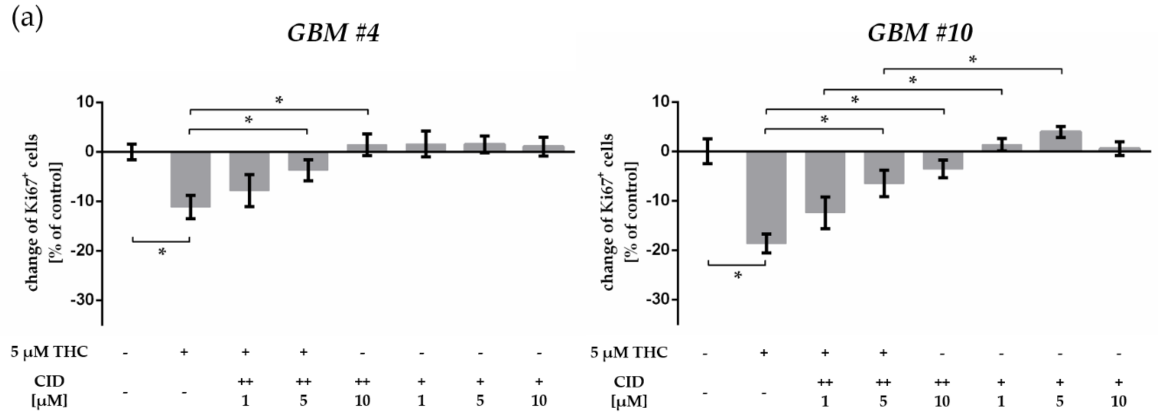


(b)

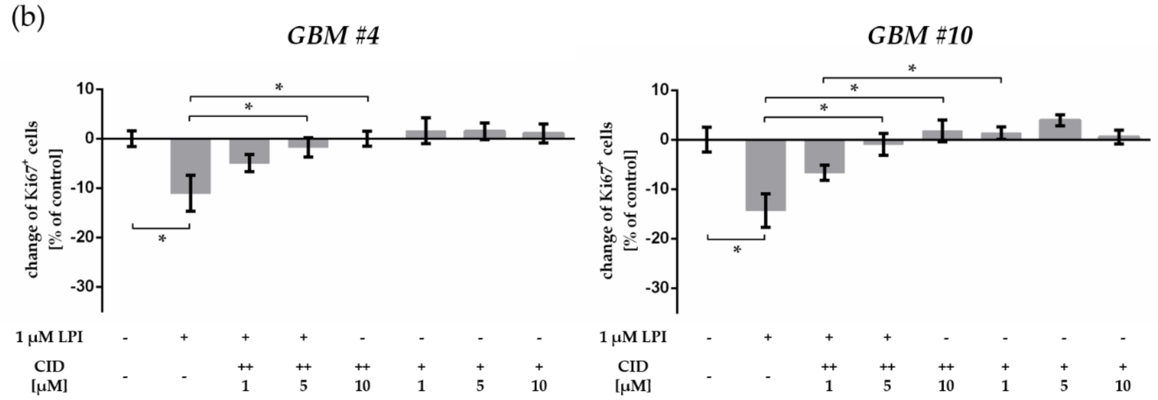


(c)

| *cell type* | *treatment* | *mean [%]* | *SEM [%]* | *sample size* |
|---|---|---|---|---|
| *GBM #4* | CTL | 0.00 | 1.56 | 6 |
| *GBM #4* | THC | -11.19 | 2.36 | 6 |
| *GBM #4* | LPI | -11.07 | 3.66 | 5 |
| *GBM #4* | 1 µM CID | 1.60 | 2.59 | 6 |
| *GBM #4* | 5 µM CID | 1.50 | 1.67 | 6 |
| *GBM #4* | 10 µM CID | 1.04 | 1.92 | 6 |
| *GBM #4* | THC + 1 µM CID | -7.85 | 2.36 | 6 |
| *GBM #4* | THC+ 5 µM CID | -3.71 | 3.25 | 6 |
| *GBM #4* | THC + 10 µM CID | 1.44 | 2.21 | 6 |
| *GBM #4* | LPI + 1 µM CID | -4.94 | 1.72 | 6 |
| *GBM #4* | LPI + 5 µM CID | -1.77 | 1.95 | 6 |
| *GBM #4* | LPI + 10 µM CID | -0.01 | 1.51 | 6 |

| *cell type* | *treatment* | *mean [%]* | *SEM [%]* | *sample size* |
|---|---|---|---|---|
| *GBM #10* | CTL | 0.00 | 2.50 | 6 |
| *GBM #10* | THC | -18.64 | 1.90 | 6 |
| *GBM #10* | LPI | -14.29 | 3.38 | 6 |
| *GBM #10* | 1 µM CID | 1.34 | 1.24 | 6 |
| *GBM #10* | 5 µM CID | 3.91 | 1.09 | 5 |
| *GBM #10* | 10 µM CID | 0.55 | 1.38 | 6 |
| *GBM #10* | THC + 1 µM CID | -12.42 | 3.21 | 6 |
| *GBM #10* | THC+ 5 µM CID | -6.49 | 2.69 | 6 |
| *GBM #10* | THC + 10 µM CID | -3.57 | 1.81 | 6 |
| *GBM #10* | LPI + 1 µM CID | -6.64 | 1.54 | 6 |
| *GBM #10* | LPI + 5 µM CID | -0.92 | 2.22 | 6 |
| *GBM #10* | LPI + 10 µM CID | 1.81 | 2.20 | 6 |

**Figure S3:** Establishment of GPR55 antagonist CID16020046 (CID). **(a)** Influence of increasing concentration of CID in presence of THC. The effects of THC were significantly abolished after pre-treatment with 5 µM or 10 µM CID in *GBM #4* and *GBM #10*. CID alone had no significant effects. **(b)** Influence of increasing concentration of CID in presence of LPI. 5 µM or 10 µM CID were sufficient to block LPI effects completely. CID alone had no significant effects. **(c)** Exact measurement values and sample sizes after treatment with increasing concentration of CID. Data are means ± SEM of N=3 independent experiments performed in duplicate. To completely block GPR55 CID were applied 15 min (++) before THC or LPI were subsequently added. Significance was chosen for $p<0.05$. The asterisk denotes significant results regarding the respective measurement indicated with the bar.

(a)

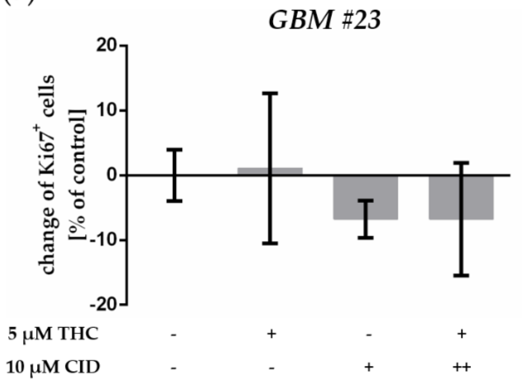


(b)

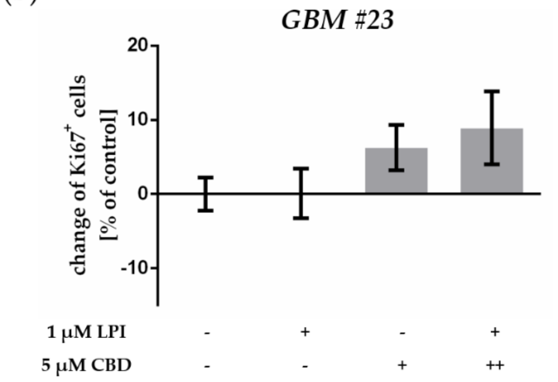


(c)

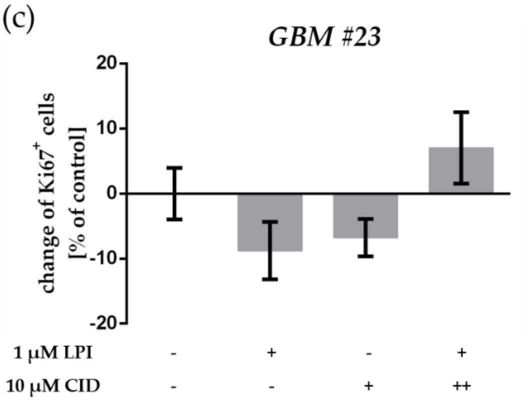


(d)

| *cell type* | *treatment* | *mean [%]* | *SEM [%]* | *sample size* |
|---|---|---|---|---|
| *GBM #23* | CTL | 0.00 | 3.97 | 5 |
| *GBM #23* | THC | 1.07 | 11.6 | 6 |
| *GBM #23* | CID | -6.77 | 2.88 | 6 |
| *GBM #23* | THC + CID | -6.77 | 8.70 | 6 |
| *GBM #23* | CTL | 0.00 | 2.22 | 6 |
| *GBM #23* | LPI | 0.80 | 3.36 | 6 |
| *GBM #23* | CBD | 6.28 | 3.06 | 6 |
| *GBM #23* | LPI + CBD | 8.95 | 4.92 | 5 |
| *GBM #23* | CTL | 0.00 | 3.97 | 5 |
| *GBM #23* | LPI | -8.81 | 4.41 | 5 |
| *GBM #23* | CID | -6.77 | 2.88 | 6 |
| *GBM #23* | LPI + CID | 7.02 | 5.48 | 6 |

**Figure S4:** Impact of GPR55 ligands on the percentage of Ki67$^{+}$ cells in *GBM #23*. **(a)** Impact of THC and CID16020046 (CID) on the number of Ki67-labelled GBM cells. Application of THC (5 μM) did not alter the percentage of Ki67$^{+}$ cells alone and in combination with CID (10 μM). CID alone displayed also no significant effects. **(b)** Impact of LPI and CBD on the number of Ki67-labelled GBM cells. Cells treated with LPI (1 μM) for 24 h were unaffected through the number of Ki67$^{+}$ cells. The combination with GPR55-antagonist CBD had also no significant effects on *GBM #23*. **(c)** Impact of LPI and CID on the number of Ki67-labelled GBM cells. Graph demonstrates the effects of CID (10 μM) alone and LPI (1 μM) in presence of CID. CID alone had no influence on the growth fraction of *GBM #23* and did not altered LPI-acting when both were co-applied. **(d)** Exact measurement values and sample sizes after treatment with $CB_1$- and $CB_2$-ligands. Data are means ± SEM of N=3 independent experiments performed in duplicate. To completely block GPR55 CBD and CID were applied 15 min (++) before THC or LPI were subsequently added.

**Table S1.** Data in patients and primary tumor samples of investigated GBM.

| | | | *GBM #4* | *GBM #10* | *GBM #23* |
|---|---|---|---|---|---|
| primary tumor sample | | sex | female | male | female |
| | | age | 50 | 56 | 83 |
| | | tumor location | temporooccipital | temporal | central |
| | | tumor origin | *de novo* | *de novo* | *de novo* |
| | | MGMT[1] promotor | methylated | methylated | unmethylated |
| | | IDH1[2] status (R132H) | wildtype | wildtype | wildtype |
| patient-derived cells | | MGMT promotor | methylated | methylated | methylated |
| | | IDH1 status (R132H) | wildtype | wildtype | wildtype |
| | stem cell marker | $CD133^+$ cells | < 1% | < 1% | < 1% |
| | | *SOX2* mRNA | detectable | detectable | detectable |
| | | *MSI1* mRNA | detectable | detectable | detectable |
| | | *NES* mRNA | detectable | detectable | detectable |
| | | *CD44* mRNA | detectable | detectable | detectable |

[1]O-6-methylguanine-DNA methyltransferase; [2] isocitrate dehydrogenase 1

**Table S2.** Exact measurement values and sample sizes after treatment with THC and CBD.

| cell type | treatment | mean [%] | SEM [%] | sample size |
|---|---|---|---|---|
| *GBM #4* | CTL | 0.00 | 2.08 | 10 |
| *GBM #4* | THC | -16.68 | 2.39 | 9 |
| *GBM #4* | CBD | -4.09 | 1.54 | 8 |
| *GBM #4* | THC + CBD | -4,75 | 3.33 | 7 |
| *GBM #10* | CTL | 0.00 | 2.27 | 7 |
| *GBM #10* | THC | -9.11 | 2.62 | 8 |
| *GBM #10* | CBD | -1.69 | 2.17 | 7 |
| *GBM #10* | THC + CBD | -0.55 | 1.78 | 6 |
| *GBM #23* | CTL | 0.00 | 2.07 | 10 |
| *GBM #23* | THC | -1.39 | 4.05 | 9 |
| *GBM #23* | CBD | 1.27 | 2.96 | 9 |
| *GBM #23* | THC + CBD | -4.55 | 2.91 | 10 |

**Table S3.** Exact measurement values and sample sizes after treatment with THC in presence of different antagonists.

| receptor | cell type | treatment | mean [%] | SEM [%] | sample size |
|---|---|---|---|---|---|
| $CB_1/CB_2$ | *GBM #4* | CTL | 0.00 | 3.17 | 12 |
| | *GBM #4* | THC | -27.66 | 1.36 | 6 |
| | *GBM #4* | AM281 + AM630 | 10.28 | 0.78 | 6 |
| | *GBM #4* | THC + AM281 + AM630 | -25.41 | 1.99 | 6 |
| | *GBM #10* | CTL | 0.00 | 3.06 | 12 |
| | *GBM #10* | THC | -21.06 | 3.08 | 6 |
| | *GBM #10* | AM281 + AM630 | 7.42 | 2.57 | 6 |
| | *GBM #10* | THC + AM281 + AM630 | -25.58 | 2.47 | 6 |
| GPR18 | *GBM #4* | CTL | 0.00 | 3.36 | 6 |
| | *GBM #4* | THC | -19.02 | 3.70 | 6 |
| | *GBM #4* | O-1918 | -7.07 | 3.39 | 6 |
| | *GBM #4* | THC + O-1918 | -19.58 | 3.13 | 6 |
| | *GBM #10* | CTL | 0.00 | 1.96 | 6 |
| | *GBM #10* | THC | -20.49 | 4.64 | 6 |
| | *GBM #10* | O-1918 | -4.118 | 3.08 | 6 |
| | *GBM #10* | THC + O-1918 | -27.95 | 3.69 | 6 |
| GPR55 | *GBM #4* | CTL | 0.00 | 1.56 | 6 |
| | *GBM #4* | THC | -11.19 | 2.36 | 6 |
| | *GBM #4* | CID | 1.04 | 1.92 | 6 |
| | *GBM #4* | THC + CID | 1.44 | 2.21 | 6 |
| | *GBM #10* | CTL | 0.00 | 2.50 | 6 |
| | *GBM #10* | THC | -18.64 | 1.90 | 6 |
| | *GBM #10* | CID | 0.55 | 1.38 | 6 |
| | *GBM #10* | THC + CID | -3.57 | 1.81 | 6 |

**Table S4.** Exact measurement values and sample sizes after treatment with increasing concentrations of THC in presence of CID.

| cell type | treatment | mean [%] | SEM [%] | sample size |
|---|---|---|---|---|
| *GBM #4* | CTL | 0.00 | 1.36 | 6 |
| *GBM #4* | 0.1 µM THC | -8.52 | 1.62 | 6 |
| *GBM #4* | 1 µM THC | -6.45 | 1.69 | 6 |
| *GBM #4* | 5 µM THC | -13.72 | 3.24 | 6 |
| *GBM #4* | 10 µM THC | -12.53 | 1.56 | 5 |
| *GBM #4* | 0.1 µM THC + CID | 1.11 | 0.76 | 6 |
| *GBM #4* | 1 µM THC + CID | -0.99 | 1.28 | 6 |
| *GBM #4* | 5 µM THC + CID | 2.21 | 1.22 | 6 |
| *GBM #4* | 10 µM THC + CID | 0.98 | 0.79 | 6 |
| *GBM #4* | CID | 0.44 | 0.61 | 5 |
| *GBM #10* | CTL | 0.00 | 3.14 | 6 |
| *GBM #10* | 0.1 µM THC | -7.04 | 1.46 | 6 |
| *GBM #10* | 1 µM THC | -8.97 | 1.95 | 6 |
| *GBM #10* | 5 µM THC | -15.48 | 3.49 | 6 |
| *GBM #10* | 10 µM THC | -16.92 | 2.19 | 5 |
| *GBM #10* | 0.1 µM THC + CID | -2.65 | 3.10 | 6 |
| *GBM #10* | 1 µM THC + CID | -280 | 2.66 | 6 |
| *GBM #10* | 5 µM THC + CID | -1.26 | 1.35 | 6 |
| *GBM #10* | 10 µM THC + CID | -5.42 | 2.4 | 6 |
| *GBM #10* | CID | 4.63 | 1.45 | 6 |

**Table S5.** Exact measurement values and sample sizes after treatment with LPI and LPI in presence of CBD and CID.

| cell type | treatment | mean [%] | SEM [%] | sample size |
|---|---|---|---|---|
| *GBM #4* | CTL | 0.00 | 2.26 | 6 |
| *GBM #4* | LPI | -13.11 | 0.82 | 6 |
| *GBM #4* | CBD | -1.70 | 1.29 | 6 |
| *GBM #4* | LPI + CBD | -1.35 | 1.75 | 6 |
| *GBM #10* | CTL | 0.00 | 3.05 | 6 |
| *GBM #10* | LPI | -13.28 | 1.72 | 6 |
| *GBM #10* | CBD | 2.81 | 0.54 | 6 |
| *GBM #10* | LPI + CBD | 3.66 | 1.76 | 6 |
| *GBM #4* | CTL | 0.00 | 1.56 | 6 |
| *GBM #4* | LPI | -11.07 | 3.66 | 6 |
| *GBM #4* | CID | 1.04 | 1.92 | 6 |
| *GBM #4* | LPI + CID | -0.01 | 1.51 | 6 |
| *GBM #10* | CTL | 0.00 | 2.50 | 6 |
| *GBM #10* | LPI | -14.29 | 3.38 | 6 |
| *GBM #10* | CID | 0.55 | 1.38 | 6 |
| *GBM #10* | LPI + CID | 1.81 | 2.20 | 6 |

**Table S6.** Exact measurement values and sample sizes after treatment with LPI and THC co-application.

| cell type | treatment | mean [%] | SEM [%] | sample size |
|---|---|---|---|---|
| *GBM #4* | CTL | 0.00 | 3.36 | 6 |
| *GBM #4* | LPI | -19.02 | 3.70 | 6 |
| *GBM #4* | THC | -15.55 | 2.78 | 5 |
| *GBM #4* | LPI + THC[1] | -18.69 | 2.60 | 6 |
| *GBM #4* | THC + LPI[1] | -19.47 | 3.15 | 6 |
| *GBM #10* | CTL | 0.00 | 1.96 | 6 |
| *GBM #10* | LPI | -20.49 | 4.64 | 6 |
| *GBM #10* | THC | -14.97 | 4.32 | 6 |
| *GBM #10* | LPI + THC[1] | -20.78 | 2.12 | 6 |
| *GBM #10* | THC + LPI[1] | -19.69 | 4.21 | 6 |

[1]15 min pre-treatment
